# Chemical Composition of Essential Oils Obtained from *Heteromorpha arborescens* (Spreng.) Cham. and Schltdl Leaves Using Two Extraction Methods

**DOI:** 10.1155/2020/9232810

**Published:** 2020-12-03

**Authors:** Taiwo Oluwafunmilola Abifarin, Gloria Aderonke Otunola, Anthony Jide Afolayan

**Affiliations:** Medicinal Plants and Economic Development (MPED) Research Centre, Department of Botany, University of Fort Hare, Alice 5700, South Africa

## Abstract

This study was aimed at comparing the essential oils obtained from *Heteromorpha arborescens* leaves by Solvent-Free Microwave Extraction (SFME) and Hydrodistillation (HD) methods in terms of their chemical compositions, yield, CO_2_ emission, and energy consumption. The solvent-free microwave extraction method indicated a higher oil yield of 0.7 mL/200 g (0.35%) as compared to 0.59 mL/200 g (0.295%) obtained through hydrodistillation. GC-MS analysis of the oils revealed a total of 52 chemical components from both methods with the presence of 35 (96.52%) and 30 (71.15%) chemical constituents for HD and SFME, respectively. The major constituents observed in the essential oil extracted by SFME methods include *α*-pinene (6%), D-limonene (11.27%), *β*-ocimene (9.09%), *β*-phellandrene (6.33%), *β*-mycene (8.49%), caryophyllene (5.96%), and camphene (4.28%). However, in the hydrodistillation method, the oil was majorly composed of *a*-pinene (4.41%), *β*-pinene (10.68%), *β*-ocimene (6.30%), germacrene-D (5.09%), humulene (5.55%), and *α*-elemene (6.18%). The SFME method was better in terms of saving energy (0.25 kWh against 4.2 kWh of energy consumed), reduced CO_2_ emission (200 g against 3360 g of CO_2_), a higher yield, and better quality of essential oil due to the presence of higher valuable oxygenated compounds (8.52%) against that of the hydrodistillation method (2.96%). The SFME method is, therefore, a good alternative for extracting the oils of *H. arborescens* leaves since the essential oil yield is higher with more oxygenated compounds, considerable energy savings, lower cost, and reduced environmental burden at substantially reduced extraction time (30 min as opposed to 180 min).

## 1. Introduction

Plants have a long history of therapeutic use in the management of diseases since time immemorial. Essential oils are natural, complex, and aromatic volatile compounds produced by plants, and they are generally present at low concentrations. These compounds possess antimicrobial and antioxidant activities, and they function in the treatment of various kinds of diseases [1]. They are also beneficial in various food industries (for preserving food against oxidation), as alternatives to artificial chemicals in cosmetics and perfumery industries for the production of various cologne waters, bathing lotions, hair lotions, and shampoos, and as components of disinfectants and insecticides [2].

Among aromatic plant species, the genus *Heteromorpha* (Apiaceae) consists of seven species which are restricted to temperate and subtropical Africa and Southern Yemen [3]. There is an increased interest for Apiaceae, especially in food science, because members of this family such as fennel, celery, dill, carrot, and caraway are commonly used. Apart from the biologically active essential oils found in this family, they contain coumarins, polyacetylenes, flavonoids, sesquiterpenes, and phthalides [4].

*Heteromorpha arborescens* (Spreng.) Cham. and Schltdl (Apiaceae) can be referred to as a large shrub, small or medium deciduous tree [5]. It is regarded as an important medicinal plant throughout its distribution area in tropical Africa and has been used for the treatment of many ailments including helminthiasis [6], abdominal pains, infertility, nervous disorders, and tuberculosis [5]. The plant is popularly known in parts of Africa for its therapeutic benefits and is therefore included in the monographic treatment of medicinal plants of South African medicine [7].

From previous studies, the volatile oil of *H. arborescens* leaves is known to contain sabinene, *δ*-3-carene, myrcene, germacrene-D, limonene, (Z)-*β*-ocimene, *β*-phellandrene, and *α*-pinene as major constituents, and it possesses both antibacterial and antifungal activities [5]. Furthermore, the essential oil of *H. arborescens*is is vital in the development of new pharmaceutical and health products in Southern Africa for headache, inhalant, and aromatherapy [8].

Various conventional methods such as hydrodistillation, steam distillation, and cold pressing have been used for essential oil extraction in aromatic plants. However, the use of these methods has been disputable for successive determination of essential oil composition because the long extraction times at high temperatures may cause changes in the essential oil composition or degradation of unsaturated or esterified compounds and the loss of highly volatile compounds [9, 10]. These limitations have led to the development of the solvent-free microwave extraction method in order to reduce the extraction time and obtain better quality of essential oils [9, 10].

The solvent-free microwave extraction (SFME) set up is referred to as a “green technology” which involves a combination of heat from the microwave and dry distillation at atmospheric pressure [11]. It is a sustainable extraction method which relies on reduced energy consumption, ensuring a safe and high quality end product [12]. It involves heating the fresh plant material in a microwave reactor, devoid of water or any solvent at all for 30 min. The short-term heating of the water in the plant material expands the plant cells causing the plant cells and oil producing glands to release the oil from the plant material [9]. SFME method has widely been employed in the extraction of oils from *Melalaica leucadendra* L. [13], *Ocimum basilicum* L. [14], *Kananga odorata* [15], *Pogostemon cabin* [16], *Cuminumcyminum* L. and *Zanthoxylum bungeanum* Maxim [17], and many others. However, until now, there has been no studies on the comparison of the quality and yield of essential oils obtained from *H. arborescens* leaves using hydrodistillation and SFME methods. This work is therefore aimed at making a comparative study in terms of extraction yields and chemical compositions of essential oils obtained from *H. arborescens* leaves using hydrodistillation and solvent-free microwave extraction methods.

### 1.1. Plant Collection

Fresh leaves of *H. arborescens* were collected in June, 2019, on a site located on latitude 32°47′50.4″ S and 26°52′41.8″ E along Hogsback road, Alice Town, Eastern Cape, South Africa. The plant was authenticated by Prof. Cupido, a taxonomist in the Department of Botany, University of Fort Hare, and a voucher specimen (Abif2019/03) was deposited in the Giffen herbarium.

### 1.2. Chemicals

Distilled water and n-hexane (chemical formular- C_6_H_14,_ MW-86.18 g/mol, BP- 68.73°C, density- 655 kg/m^3^, and purity-95%) used in the study were of analytical grade.

## 2. Methodology

### 2.1. Determination of Moisture Content

200 g of the fresh *H. arborescens* leaves was weighed and recorded as *W*_*f*_, dried at 80°C for 72 hours, and the dried leaves were then weighed and recorded as W_d_. The moisture content of the leaves was calculated as

(1) $$\begin{array}{c} \text{Moisture content}\left(\%\right)=\frac{W_{f}W_{d}}{W_{f}}\times 100. \end{array}$$

### 2.2. Extraction by Hydrodistillation

200 g of fresh *H. arborescens* leaves was subjected to hydrodistillation with a Clevenger-type apparatus as described by the European Pharmacopoeia and extracted with 1L of water for 180 min at 100°C. The essential oil was collected and analyzed by GC-MS.

### 2.3. Solvent-Free Microwave Extraction (SFME)

200 g of fresh *H. arborescens* leaves was placed into the reactor without addition of any solvent. Microwave extraction was performed using a Milestone MAO20-A apparatus; a multimode microwave reactor 2.45 GHz with a maximum delivered power of 500 W variable in 5 W increments. The essential oil was completely extracted at atmospheric pressure and 99.85°C (373 K) within 30 min; collected, and subjected to GC-MS analysis.

### 2.4. Energy Consumption and CO_2_ Emission

Power consumption was 500 W and 1400 W for SFME and hydrodistillation methods, respectively. The energy consumption and CO_2_ emission were calculated according to previous literature as energy consumption (kWh) = Pt/1000 and to obtain 1 kWh from the combustion of coal or fossil fuel, and 800 g of CO_2_ will be emitted into the atmosphere [18–20].

### 2.5. Extraction Yields

The extraction yields of the essential oils obtained from both methods were calculated as

(2) $$\begin{array}{c} \text{Extraction yield}\left(\%\right)=\frac{\text{Mass of extracted oil}}{\text{Mass of Fresh Leaves}}\times 100. \end{array}$$

### 2.6. Gas Chromatography-Mass Spectroscopy Analysis

The essential oils extracted were separately analyzed by gas chromatography-mass spectroscopy (Agilent 6890 GC, coupled to an Agilent 5975 mass spectrometric detector). Gas chromatography-mass spectrometry was combined with a chromatography column HP-5.5% phenylmethylsiloxane, with 30 m length, 0.32 mm film thickness, 0.25 *μ*m internal diameter. The injection port was held at 230°C, while the interface was at 280°C. The temperature was set from 50°C to 280°C at 10°C per minute, using helium as the carrier gas.

The chemical components of the essential oils were identified by comparing their mass spectra and retention indices with those in PubChem, NIST, and Wiley libraries [21]. The spectrogram of each identified compound was determined by integration of the peak areas.

## 3. Results

The moisture content of the 200 g of *H. arborescens* leaves was 10.20 ± 0.95%, and a slightly higher oil yield of 0.7 mL/200 g (0.35%) was obtained through SFME after 30 min of extraction as compared to 0.59 mL/200 g (0.295%) obtained after 180 min by the hydrodistillation method. The oils were pale-yellow liquids with fine aroma. For the energy consumption and environmental footprint, the amount of energy consumed and the quantity of carbon dioxide emitted into the atmosphere were higher for the hydrodistillation method (4.2 kWh and 3360 g CO_2_/g of essential oil) as compared to the SFME method (0.25 kWh and 200 g CO_2_/g of essential oil).

A total of 52 compounds obtained by hydrodistillation and solvent-free microwave extraction methods are summarized in Table 1. Thirty-five compounds representing 96.52% of the total essential oil present were obtained in the hydrodistillation method. More specifically, the dominant compounds present in the hydrodistilled oil were D-limonene (11.27%), *β*-ocimene (9.09%), D-germacrene (8.92%), *β*-myrcene (8.49%), and humulene (5.55%). The SFME method, on the other hand, gave 30 compounds which accounted for 71.15% of the total oil, while *β*-pinene (10.38%), *γ*-elemene (6.18%), and D-germacrene (5.09%) were the major components. Thirteen (13) of the total components were common to oils extracted by both methods.

## 4. Discussion

Some conventional methods used for the extraction of essential oils are hydrodistillation, steam distillation, Microwave-Assisted Extraction (MAE), and solvent extraction. Although these techniques have been used over time, they have some limitations such as longer time of extraction, low extraction effectiveness, reduced oil quality due to degradation of unsaturated or ester compounds through thermal or hydrolytic effects, and negative impacts on the environment [22]. SFME and Microwave Hydrodiffusion and Gravity (MHG) methods are two new green techniques employed in the extraction of quality essential oils at a lower cost, reduced time, and environmental footprints [23].

The chemical compositions of the essential oil of *H. arborescens* leaves obtained by the solvent-free microwave extraction method have been compared with those which were obtained by the hydrodistillation method. Our study indicates that the essential oil yield obtained by the SFME method was slightly higher with shorter extraction time (0.35% for 30 min) when compared with that by the hydrodistillation method (0.295% for 180 min). The lower yield obtained by the hydrodistillation method may be attributed to increasing extraction time [24]. The present study also revealed that the SFME method reduced energy consumption and CO_2_ emission by about 16 times. Similar observations were made in previous studies [25, 26].

The two oils were characterized with monoterpene hydrocarbons, sesquiterpenes, and oxygenated compounds. However, monoterpenes and sesquiterpenes were dominant in the SFME and hydrodistilled oils, respectively, but higher quantity of oxygenated compounds was present in the SFME-extracted oil. This is in agreement with the essential oil compositions of *Angelica sylvestris* L. var. sylvestris fruits [27], *Ferula glauca*, *Ferula arrigonii*, and *Ferula communis* [28], all members of the Apiaceae family. Lower quantity of monoterpenes with higher sesquiterpenes and oxygenated compounds was observed in the SFME-derived oil. Monoterpene hydrocarbons are less important than oxygenated compounds in terms of their impact on the pleasant aroma of essential oils, while the oxygenated compounds are highly fragrant and, hence, the most important [26]. Nevertheless, monoterpenes are generally useful for therapeutic purposes as antibacterial and antifungal agents [29]. Sesquiterpenes also possess anti-inflammatory and anticarcinogenic properties [1].

Higher oxygenated compounds observed in the solvent-free microwave-extracted oil indicate the superiority of the method over hydrodistillation and could be attributed to reduced water content in the system which would have minimized the thermal and hydrolytic degradation of oxygenated compounds when compared with the hydrodistillation method [30, 31].

The major constituents observed in the essential oil extracted by the SFME method include *a*-pinene (6%), D-limonene (11.27%), *β*-ocimene (9.09%), *β*-phellandrene (6.33%), *β*-mycene (8.49%), caryophyllene (5.96%), and camphene (4.28%). However, in the hydrodistillation method, the oil was majorly composed of *α*-pinene (4.41%), *β*-pinene (10.68%), *β*-ocimene (6.30%), germacrene-D (5.09%), humulene (5.55%), and *a*-elemene (6.18%). This is in correlation with the findings of Mwangi et al. [32] and Chagonda et al. [33] in their observations that sabinene, *δ*-3-carene, myrcene, germacrene-D, limonene, (Z)-*β*-ocimene, *β*-phellandrene, and *α*-pinene are major constituents in *H. arborescen*s leaves. The absence of sabinene and *δ*-3-carene in this study could be as a result of the difference in their geographic areas or some biotic and biotic factors in the environment [34]. It is interesting to note that 22 compounds were unique to only the hydrodistillation method and 17 were distinctive to the SFME methods, while 13 were common to both methods. This buttresses the point that the chemical composition of essential oils is dependent on the extraction methods [35].

## 5. Conclusions

This study has shown that the chemical composition of the essential oils obtained from *H.arborescens* leaves is dependent on the extraction method. The SFME method resulted in a higher yield in terms of quantity and an essential oil of better quality due to the presence of higher valuable oxygenated compounds than that which was obtained by hydrodistillation. Furthermore, the use of the SFME method indicated lower energy consumption and CO_2_ emission. It can therefore be concluded that the SFME method is considered as a good alternative for the extraction of essential oil from *H. arborescens* leaves at shorter extraction time, reduced energy consumption, lower cost, and environmental footprint when compared with the hydrodistillation method.

## Figures and Tables

**Table 1:** Chemical constituents of essential oils obtained from *H. arborescens* leaves by hydrodistillation and solvent-free microwave extraction methods.

S/N	Chemical compounds	Class of compounds	KI	RT	HD (%)	SFME (%)
1	3-Thujene	MH	932	3.805	0.21	—
2	*α*-Pinene	MH	936	3.893	6.0	4.41
3	Camphene	MH	948	4.028	4.28	0.58
4	2-Thujene	MH	952	4.195	2.33	—
5	*β*-Myrcene	MH	983	4.293	8.49	—
6	*α*-Phellandrene	MH	998	4.463	6.33	1.78
7	3-Carene	MH	1002	4.509	1.88	—
8	D-limonene	MH	1027	4.658	11.27	—
9	Β-ocimene	MH	1050	4.771	9.09	6.30
10	Β-pinene	MH	973	4.236	—	10.68
11	(−)-Myrtenal	MH	1488	5.998	0.72	—
12	o-Cymene	SH	1011	5.376	—	0.42
13	*α*-Copaene	SH	1390	7.314	3.30	2.30
14	*β-*cymene	SH	1026	5.038	1.31	0.47
16	Caryophyllene	SH	1419	7.644	5.96	3.64
17	*β*-Copaene	SH	1102	7.691	2.77	0.44
18	*β*-Gurjurene	SH	1089	7.393	—	1.42
19	Γ-Terpinene	MH	987	4.896	1.30	—
20	(+)-4-Carene	MH	998	5.127	3.80	—
21	Aromandendrene	SH	1106	7.772	0.31	1.52
22	*α*-Humulene	SH	1446	7.869	5.55	—
23	*γ*-Muurolene	SH	1442	7.967	—	2.38
24	D-germacrene	SH	1481	8.035	8.92	5.09
25	*γ*-Elemene	SH	1508	8.127	—	6.18
26	*β*-Cadinene	SH	1126	8.246	2.24	3.98
27	*α*-Calacorene	SH	1133	8.406	—	1.12
28	(-)-Palustrol	OM	1141	8.603	—	0.96
30	(-)4-Terpinol	OM	1182	5.837	0.69	—
31	*α*-Terpineol	OM	1197	5.920	0.30	—
32	Spatulenol	OS	1530	8.646	—	1.78
33	3,4-Nonadiene	MH	1048	6.143	0.16	—
34	Geraniol	OM	1065	6.319	0.10	—
35	Caryophyllene oxide	OS	1574	8.696	—	2.0
36	Ledol	OM	1148	8.751	—	2.77
37	Ledene	SH	1151	8.820	—	1.64
38	Isoledene	SH	1375	7.795	0.93	—
39	*β*-Cubenene	SH	1470	8.329	0.12	—
40	Nerodiol	OS	1133	8.407	0.87	—
41	*β*-Selinene	SH	1143	8.638	0.10	—
43	Napthalene	SH	1155	8.917	0.34	0.74
44	*α-*Cardinol	OM	1161	9.063	0.24	—
45	2-Isopropyl-5-methyl-9-methylene[4,4.0]dec-1-ene	SH	1159	8.990	0.41	—
46	Phytol	OD	1949	11.289	0.63	—
47	*α*-Himachalene	SH	1158	8.983	—	1.98
48	*γ*-Gurjunene	SH	1163	9.092	—	1.57
49	Valencene	SH	1410	2.09	—	2.09
50	*α*-Bulnesene	SH	1240	10.889	—	1.16
51	Kaur-16-ene	OD	1214	10.994	—	0.91
52	D-Citral	OM	368	10.563	—	0.10
	Monoterpene hydrocarbons				57.17	23.75
	Sesquiterpenes				36.29	38.64
	Oxygenated compounds				2.96	8.52

MH- monoterpenes hydrocarbons, SH- sesquiterpenes hydrocarbons, OD- oxygenated diterpenes, OM- oxygenated monoterpenes, OS- oxygenated sesquiterpenes, KI- Kovatz Index, RT- retention time.

## Data Availability

All underlying data supporting the results can be found in the manuscript.
